# Short-Term Speed Variability as an Index of Pacing Stochasticity in Athletic Running Events

**DOI:** 10.3390/jfmk8020086

**Published:** 2023-06-19

**Authors:** Daniel Boullosa, Eliésdras Patrocínio, Andrew Renfree, Arturo Casado, Brian Hanley, Carl Foster

**Affiliations:** 1 Faculty of Physical Activity and Sports Sciences, Universidad de León, 24007 León, Spain; 2 Graduate Program in Movement Sciences, Federal University of Mato Grosso do Sul, Campo Grande 79070-900, Brazil; esdraspatrocinio@gmail.com; 3 College of Healthcare Sciences, James Cook University, Townsville 4811, Australia; 4 Institute of Sport and Exercise Science, University of Worcester, Worcester WR2 6AJ, UK; a.renfree@worc.ac.uk; 5 Centre for Sport Studies, Rey Juan Carlos University, 28933 Madrid, Spain; arturo.casado@urjc.es; 6 Carnegie School of Sport, Leeds Beckett University, Leeds LS6 3QS, UK; b.hanley@leedsbeckett.ac.uk; 7 Department of Exercise and Sports Science, University of Wisconsin-La Crosse, La Crosse, WI 54601, USA; cfosteruwl@gmail.com

**Keywords:** aerobic endurance, aerobic performance, endurance running, athletics

## Abstract

We aimed to compare differences in performance and pacing variability indices between 5000 m heats and finals during major championships in men and women. Data with 100 m time resolution were used to compare overall pacing variability (standard deviation of 100 m section times, SD; and coefficient of variation, CV%) and short-term pacing variability (root mean square of successive differences between 100 m section times, RMSSD). The changes in performance and pacing indices differed between races and competitions. For instance, the men’s final in Beijing 2008 was quicker than the heat (*p* < 0.01) while the CV% was reduced (*p* = 0.03) and RMSSD increased (*p* < 0.01). For women, the heats and the final exhibited a similar mean time in London 2017 (*p* = 0.33) but with CV% (*p* < 0.001) and RMSSD (*p* < 0.001) showing opposite trends. Individual analyses of men’s and women’s champions revealed highly individual variability metrics. The use of RMSSD can complement overall variability indices for better characterization of pacing stochasticity.

## 1. Introduction

Pacing can be defined as the distribution of work over a time or distance trial. In track and field, the past 30 years of research have demonstrated that pacing strategies differ depending on race distance and its contextual factors (e.g., heats vs. finals) [1,2]. Effective pacing is a prerequisite for success as more even pacing strategies are associated with faster performances in endurance athletic events. However, when the same athlete succeeds in breaking their own personal record, there is little change in pacing behavior [3]. Thus, we need more sensitive methods to better explain such little changes in pacing and subsequent performances for a better evaluation of pacing behaviors.

The most frequently used methods to analyze pacing behavior include the comparison of the mean speed between the different race segments (e.g., tertiles or quartiles of the total distance) and coefficient of variation (CV%) of speed [1,4]. These methods capture the overall pacing variability around a mean speed. However, tactical demands in competitive races impose a high degree of decision-making, leading to frequent speed variations [4] (e.g., brief accelerations and decelerations) that are especially pronounced during championship qualifying rounds and finals [5]. These changes in speed may be different between sexes [6]. They can also determine the potential for success of an athlete depending on the D’ balance (i.e., how much an athlete runs below/over the critical speed) [7]. These pacing micro-variations or short-term pacing variability are not captured by traditional methods (e.g., CV%) of pacing analyses. In this regard, simple calculations such as the root mean square of successive differences of time series (RMSSD) could be appropriate to assess the changes in mean speed between adjacent race segments. The usefulness of this algorithm is well recognized in physiology since it represents the most robust short-term variability analysis of heart rate (HR) which shows the instantaneous vagal influence on the HR [8]. Consequently, the mathematical properties of RMSSD could be used to better evaluate short-term variability of velocity/speed as it is used for the same purpose with HR.

Therefore, the aim of the current exploratory study is to calculate both overall (i.e., SD, CV%) and short-term pacing variability (i.e., RMSSD) in men’s and women’s track running races to verify whether they provide different information when analyzing pacing characteristics during highly stochastic races of major championships. This new procedure will be useful for a better evaluation of athletes’ pacing stochasticity.

## 2. Materials and Methods

Publicly available data from World Athletics databases were used. As this is an exploratory study, we compared the performances of athletes participating in both the heats and the finals of various 5000 m major championships. As segment times were recorded every 100 m, we obtained 50 time series per athlete in each race. The races included were the men’s heats and finals from the Beijing 2008 Olympic Games and the London 2017 World Athletics Championships; and the women’s heats and finals from the Rio 2016 Olympic Games and the London 2017 World Athletics Championships. The main reasons for including these races were their publicly available data and that the number of participants of both sexes completing both the heats and finals were similar (14 men vs. 15–17 women).

### Statistical Analyses

The mean ± SDs of all individual time series were computed in Excel. Subsequently, the coefficient of variation (CV%; [SD/mean] ∗ 100) was calculated. The RMSSD between 100 m section times were also computed in Excel. Comparisons between heats and finals at the same major championship were performed with a paired *t*-test. The *p* value was set at 0.05.

## 3. Results

The comparisons of both overall (SD, CV%) and short-term (RMSSD) variability analyses between heats and finals in Beijing 2008 and London 2017 for men and in Rio 2016 and London 2017 for women are presented in Table 1 and Table 2, respectively.

**Figure 1:**
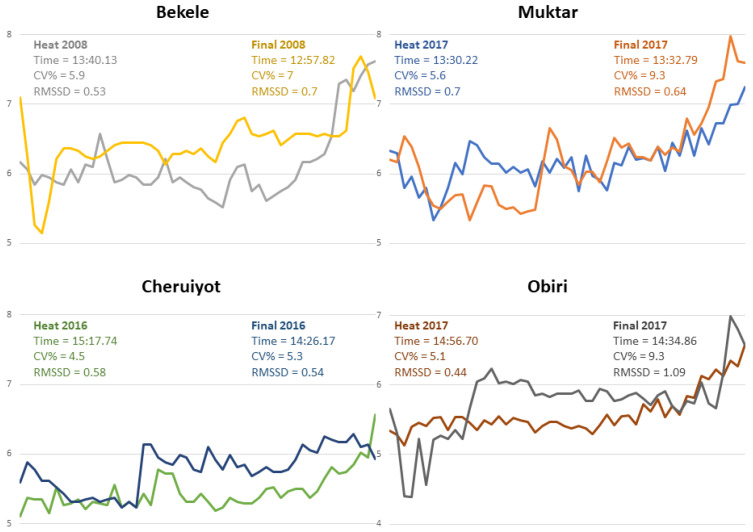
Pacing behaviors of winners of both sexes (Bekele and Muktar ♂, Cheruiyot and Obiri ♀) during the heats and finals analyzed. CV%: coefficient of variation; RMSSD: root mean square of successive differences of 100 m section times (s).

The pacing profiles and their respective metrics with a 100 m resolution of men’s and women’s champions in both heats and finals are presented in Figure 1.

## 4. Discussion

This is an exploratory study analyzing overall and short-term pacing variability in different major championships in both men and women, aiming to determine whether there are differences between variability metrics. As expected, the mean values of both overall and short-term pacing variability revealed different trends between heats and finals when analyzing race performances characterized by substantially different tactical behaviors and mean speeds. In addition, when examining the variability metrics of men’s and women’s champions, it is evident that there is not a unique pattern of pacing behavior with races of similar times showing different variability metrics and vice versa. More importantly, changes in CV% are not necessarily linked to changes in RMSSD. For instance, Muktar recorded similar finishing times in the men’s heat and final in London 2017. However, in the final, the overall variability increased (i.e., CV%) while short-term variability (i.e., RMSSD) decreased.

These results suggest a great potential of RMSSD to better analyze and understand pacing behavior in competitive settings as it captures changes between adjacent time sections whereas the CV% assesses only the variability around the mean speed or time. For instance, while previous evidence revealed a reduced CV% when different runners broke World Records, a new analysis of World Records broken by the same athlete revealed no changes in CV% [3]. The use of RMSSD is therefore helpful in this context to identify whether some sudden surges or decelerations throughout the race are related to differences in performance that are not evident through the analysis of CV%. In this regard, the calculation of RMSSD for the comparison of race sections (e.g., halves, tertiles) could also be pertinent in some cases to better identify what race sections are more stochastic.

One important aspect to be considered refers to the resolution used in the current study (i.e., 100 m section times) as sudden accelerations and decelerations might impact this metric more significantly with shorter sections than the current ones. Furthermore, other complexity measures such as fractal [9,10] and entropy [11] analyses would even require a greater frequency of time series for appropriate validity. Therefore, further studies should identify the optimal resolution of time series needed to capture the influence of sudden surges and decelerations while allowing valid complexity analyses. This necessity has to also consider the specific characteristics of using different technologies on the track (e.g., WaveLight pacing) and the field (e.g., GPS). Meanwhile, it should be pointed out that RMSSD (a) may not accurately capture short-term variability if data series are too short; (b) is influenced by extreme values or outliers which can have a disproportionate impact on its values; (c) does not present an accepted threshold or benchmark for what constitutes high or low values; (d) might present a sex bias during its use. Future investigations should address these issues to assist in the identification of the real potential of this metric to capture pacing stochasticity in both sexes.

The use of this new measure of pacing stochasticity is very simple as its value can be easily calculated with an Excel spreadsheet when a minimum of time series of running speed is available. Of note, the use of these pacing metrics can be extended to endurance sports other than running. For the individual athlete, coaches can calculate both the CV% and RMSSD of speed in different races over the same distance during the season to check whether there are changes in pacing with respect to the mean speed with the use of CV%, and whether there are changes in stochasticity (e.g., less steady pace because of tactical decisions) with the use of RMSSD. These analyses can be complemented with qualitative data considering the level of the opponents or the importance of the race in the context of the competitive season. Exploratory analyses of race subsections and laps with the support of pacing graphical profiles can also be considered to better understand the impact of specific tactical decisions on pacing behaviors. More specifically, the combined use of RMSSD while knowing the critical speed and D’ of an athlete would allow a better characterization of pacing behaviors with respect to their endurance running capacity and potential for improvement. Further, these analyses are not exclusive to competitive settings as CV% and RMSSD calculations can be also derived from training speed recordings to identify pacing behaviors during race simulations or interval training sessions with and without the presence of other athletes. This may be relevant in the context of endurance sports, in which the coach is not always present during training sessions, to better understand what happens during specific training sessions with the analysis of speed recordings. Finally, the combination of these pacing metrics could be also applied to time or distance trial testing to increase the sensitivity of pacing metrics to verify the concurrent evolution of physiological adaptations and pacing behaviors of athletes over time. In this context, RMSSD may be also helpful for biomechanical testing of runners in the field to check the steadiness of running pace at different speeds when the speeds of the sections evaluated are not imposed by an external cue.

## 5. Conclusions

Short-term (RMSSD) and overall (SD and CV%) variability indices captured different within-championship pacing characteristics for men and women during highly stochastic races. The use of RMSSD is recommended to complement overall variability indices for a better characterization of pacing stochasticity.

The use of RMSSD would be helpful to compare within- and between-athlete performances over the same or different distances to better characterize short-term variability in pacing that likely reflects the degree of sudden accelerations and decelerations occurring because of tactical behaviors of competitive athletes. Although we used the available data with a resolution of 100 m section times, the use of higher resolutions would be desirable for this purpose in future analyses. The use of RMSSD could be also extended to other contexts including training and evaluation settings in different endurance sports.

## Figures and Tables

**Table 1:** Mean ± SD of final times, overall (SD and CV%) and short-term (RMSSD) pacing variability of heats and finals in men’s races.

Race—Championship (Day, Time, Temperature)	Time (s)	*p* Values and Trends	SD	*p* Values and Trends	CV%	*p* Values and Trends	RMSSD	*p* Values and Trends
Heat—Beijing (20 August 2008, 8:27 h, ~24 °C)	822 ± 4	<0.001 ↓	0.88 ± 0.04	<0.001 ↓	6.96 ± 1.28	0.03 ↓	0.55 ± 0.06	<0.001 ↑
Final—Beijing (23 August 2008, 20:10 h, ~24 °C)	802 ± 15		0.63 ± 0.15		5.9 ± 0.61		0.69 ± 0.04	
Heat—London (9 August 2017, 20:05 h, ~14 °C)	806 ± 4	<0.001 ↑	0.57 ± 0.10	<0.001 ↑	4.77 ± 0.83	<0.001 ↑	0.63 ± 0.10	0.12 ↑
Final—London (12 August 2017, 20:20 h, ~19 °C)	819 ± 7		1.08 ± 0.12		8.13 ± 1.1		0.69 ± 0.06	

CV%: coefficient of variation; RMSSD: root mean square of successive differences of 100 m section times (s).

**Table 2:** Mean ± SD of final times, overall (SD and CV%) and short-term (RMSSD) pacing variability of heats and finals in women’s races.

Race—Championship (Day, Time, Temperature)	Time (s)	*p* Values and Trends	SD	*p* Values and Trends	CV%	*p* Values and Trends	RMSSD	*p* Values and Trends
Heat—Rio (17 August 2016, 10:05 h, ~24 °C)	926 ± 24	<0.001 ↓	0.68 ± 0.28	0.07 ↓	4.06 ± 1.01	0.08 ↑	0.72 ± 1.04	0.41 ↓
Final—Rio (20 August 2016, 21:30 h, ~22 °C)	905 ± 29		0.60 ± 0.17		5.31 ± 3.21		0.50 ± 0.09	
Heat—London (10 August 2017, 19:30 h, ~20 °C)	900 ± 3	0.33 ↓	0.70 ± 010	<0.001 ↑	8.18 ± 0.81	<0.001 ↓	0.66 ± 0.33	<0.001 ↑
Final—London (13 August 2017, 20:35 h, ~21 °C)	896 ± 13		0.96 ± 0.08		5.43 ± 0.78		1.06 ± 0.07	

CV%: coefficient of variation; RMSSD: root mean square of successive differences of 100 m section times (s).

## Data Availability

The data that support the findings of this study are available from Brian Hanley upon reasonable request.
